# Acute Lupus Pneumonitis and Severe Multisystem Complications: A Complex Inpatient Course

**DOI:** 10.7759/cureus.95320

**Published:** 2025-10-24

**Authors:** Annabelle Milorde Attolico, Zubair Khalid, Deepak Ramachandran, Ayobami B Omodara, Mehadi Hasan

**Affiliations:** 1 Internal Medicine, Mid and South Essex NHS Foundation Trust, London, GBR; 2 Respiratory Medicine, Southend Hospital, Southend-on-Sea, GBR; 3 Rheumatology, Mid and South Essex NHS Foundation Trust, London, GBR; 4 Cardiology, Southend Hospital, Southend-on-Sea, GBR; 5 Internal Medicine, Manchester University NHS Foundation Trust, Manchester, GBR

**Keywords:** acute lupus pneumonitis, heart failure, immunosuppression therapy, multisystem involvement, systemic lupus erythema

## Abstract

Systemic lupus erythematosus (SLE) is a chronic autoimmune disease with diverse systemic manifestations. Pulmonary involvement is common, but acute lupus pneumonitis (ALP) is rare, occurring in fewer than 5% of patients, and is associated with poor outcomes. We describe a 60-year-old woman with longstanding SLE/Sjögren’s overlap who presented with progressive dyspnea, pleuritic chest pain, and productive cough. Radiology revealed bilateral ground-glass opacities consistent with ALP. Her clinical course was complicated by superimposed infection, severe left ventricular dysfunction with decompensated heart failure, stage 2 acute kidney injury, and chronic small vessel cerebrovascular disease. She required intensive immunosuppression, antimicrobial therapy, and advanced cardiac support, with eventual stabilization and discharge. This case illustrates the diagnostic challenges of distinguishing ALP from infection and cardiogenic pulmonary oedema, the interplay of multisystem disease in SLE, and the importance of early multidisciplinary involvement in critically ill patients.

## Introduction

Systemic lupus erythematosus (SLE) is a systemic autoimmune disorder characterized by autoantibody production and multi-organ inflammation. Pulmonary involvement occurs in up to half of patients, with manifestations ranging from pleuritis to interstitial lung disease, alveolar hemorrhage, and acute lupus pneumonitis (ALP) [1-3]. ALP is uncommon (<5% of SLE cases) but carries a mortality rate exceeding 40-50% in some series [4,5].

Given its rarity and overlapping features with bacterial pneumonia and cardiogenic pulmonary edema, prompt recognition is critical for patient survival [2]. Differentiating ALP from cardiogenic pulmonary edema can be difficult, as both can present with bilateral infiltrates and respiratory distress. However, ALP is typically characterized by ground-glass opacities on imaging, normal or mildly elevated cardiac filling pressures, and minimal response to diuretics, whereas cardiogenic edema is associated with elevated natriuretic peptides, cardiomegaly, and rapid improvement with diuresis [6].

We present a rare and diagnostically challenging case of ALP in a patient with concomitant severe systolic heart failure and renal impairment, illustrating the complex interplay between autoimmune inflammation, cardiac dysfunction, and infection risk in SLE. 

## Case presentation

Patient history

A 60-year-old woman with a history of SLE/Sjögren’s overlap (diagnosed in 2010, anti-nuclear antibody (ANA) >1:640, anti-dsDNA, anti-Sm, and anti-cyclic citrullinated peptide (CCP) antibodies, with discoid rash and sicca symptoms) was admitted with five weeks of progressively worsening dyspnoea, pleuritic chest pain, exertional discomfort, reduced mobility, and productive cough. She denied any recent fevers, alopecia, aphthous ulcers, arthralgia, or myalgia. Urine dip was negative for protein.

Her past medical history included lupus nephritis (class V, biopsy 2022), chronic kidney disease (stage 3), non-ST elevation myocardial infarction with left anterior descending (LAD) stenting (2023), with the last echo in this year showing moderate aortic stenosis, left ventricular hypertrophy with ejection fraction (EF) of 45%, and mild pericardial effusion. She was maintained on hydroxychloroquine and mycophenolate mofetil.

Clinical findings

On admission, she presented with a saturation drop of 66%, requiring 4 L of oxygen with bilateral diffuse coarse crackles. She was afebrile, normotensive, and tachypneic with a respiratory rate of 25/min.

Diagnostic assessment

**Figure 1:**
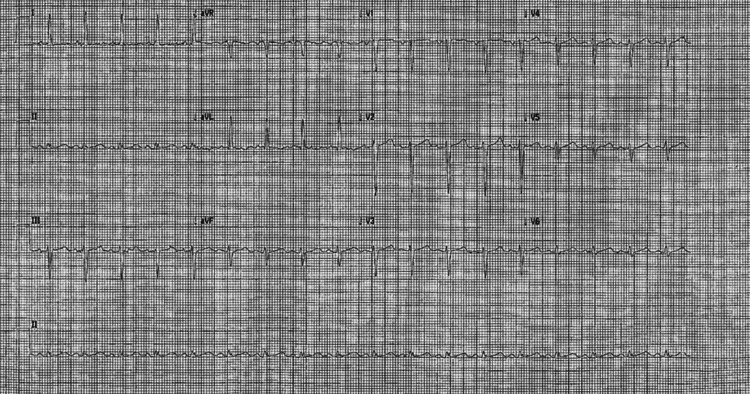
ECG showed a heart rate of 84 bpm with sinus rhythm, lateral T-wave inversion.

ECG (Figure 1) showed a heart rate of 84 bpm with sinus rhythm, lateral T-wave inversion; troponin T rose from 16 to 22 ng/L.

**Figure 2:**
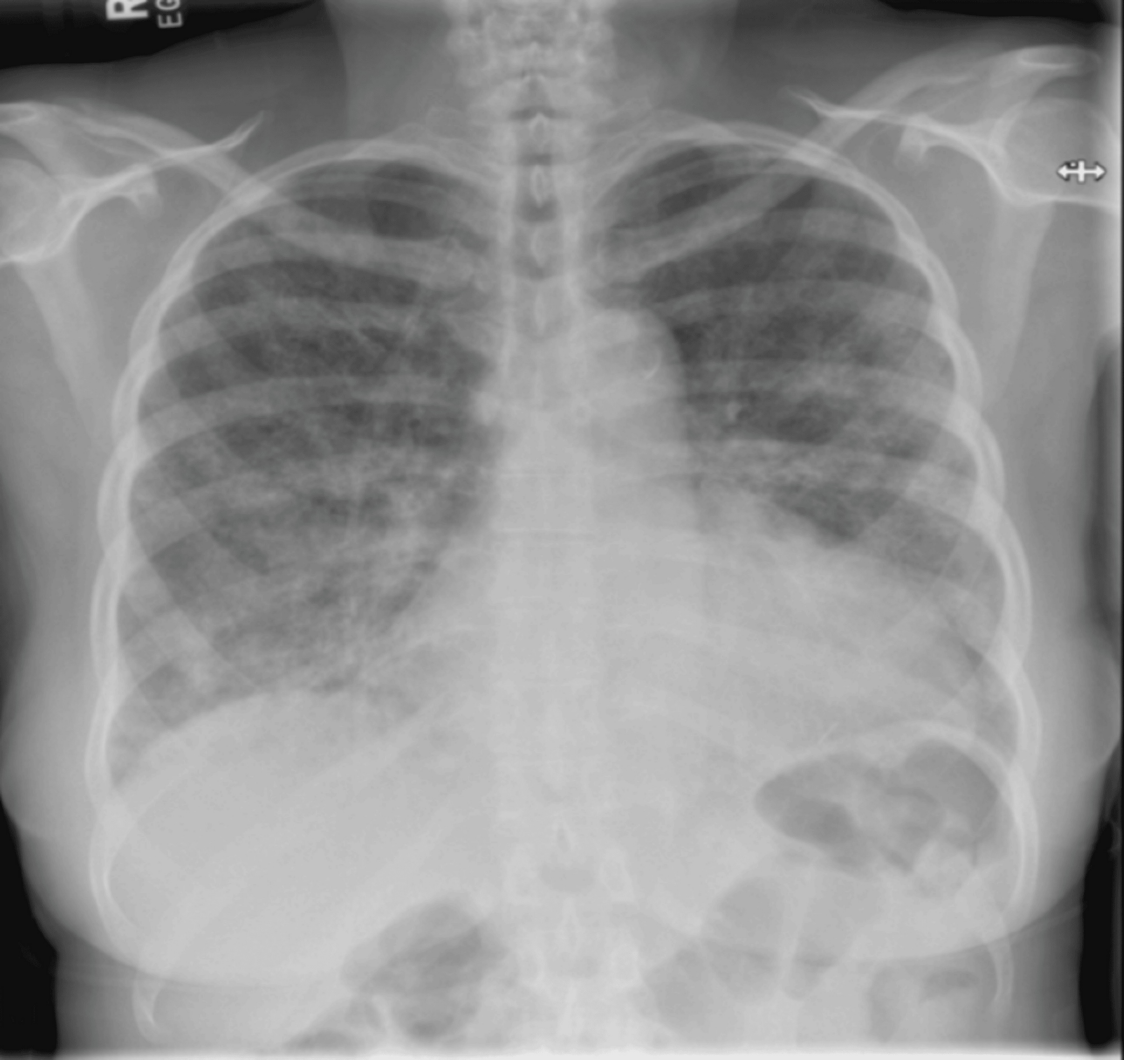
Chest X-ray on admission demonstrating bilateral patchy airspace opacities and areas of ground-glass attenuation, more prominent in the lower zones. No pleural effusion, or pneumothorax is identified. The cardiomediastinal silhouette is within normal limits. No acute osseous abnormality is seen.

Subsequently, a chest radiograph (Figure 2) was done, which revealed cardiomegaly and diffuse patchy opacities.

Her blood tests revealed CRP of 23 mg/L, haemoglobin (Hb) of 90 g/L, white cell count (WCC) of 6.0 x 10^9^/L, lymphocyte count of 0.57 x 10^9^/L (Table 1), normal liver, and renal function tests, as well as complement C4 of 0.47 g/L and complement C3 of 1.29 g/L. The procalcitonin (PCT) came normal (0.07 ug/L), with a urine protein creatinine ratio (PCR) of 29.5 mg/mmol. Blood and urine cultures were negative. Viral PCR was negative for any infection.

**Table 1:** Laboratory findings on admission.

Laboratory Tests	Values	Reference Range
Hb	90 g/L	115–160 g/L (female)
WCC	6.0 x 10⁹/L	4.0–11.0 x 10⁹/L
Lymphocytes	0.57 x 10⁹/L	1.0–3.5 x 10⁹/L
CRP	23 mg/L	<5 mg/L
Procalcitonin	0.07 µg/L	<0.1 µg/L
Troponin T	16 → 22 ng/L	<14 ng/L
Urine Protein Creatinine Ratio	29.5 mg/mmol	<15 mg/mmol (normal); 15–45 mg/mmol (microalbuminuria)
Complement C3	1.29 g/L	0.75–1.65 g/L
Complement C4	0.47 g/L	0.14–0.54 g/L

**Figure 3:**
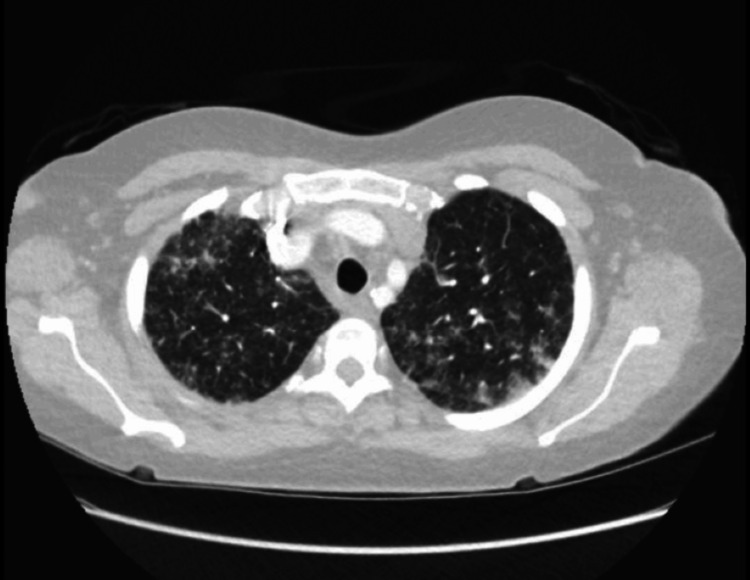
The apical region on CTPA (lung attenuation) shows bilateral patchy ground-glass opacities and areas of interlobular septal thickening, predominantly in the peripheral and peribronchovascular regions. These findings are consistent with acute lupus pneumonitis.

**Figure 4:**
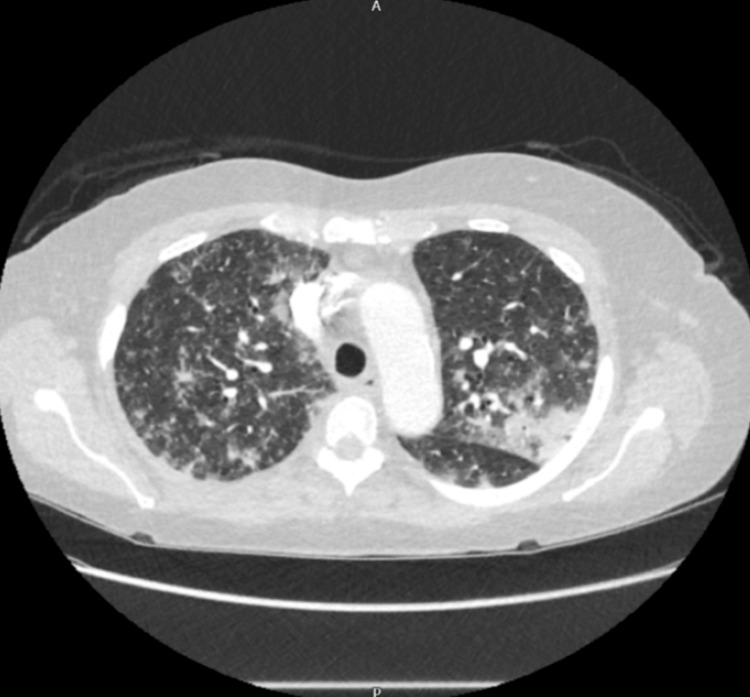
CTPA showing patchy bilateral consolidation and inflammatory changes at the mid zone level. CTPA: CT pulmonary angiography

**Figure 5:**
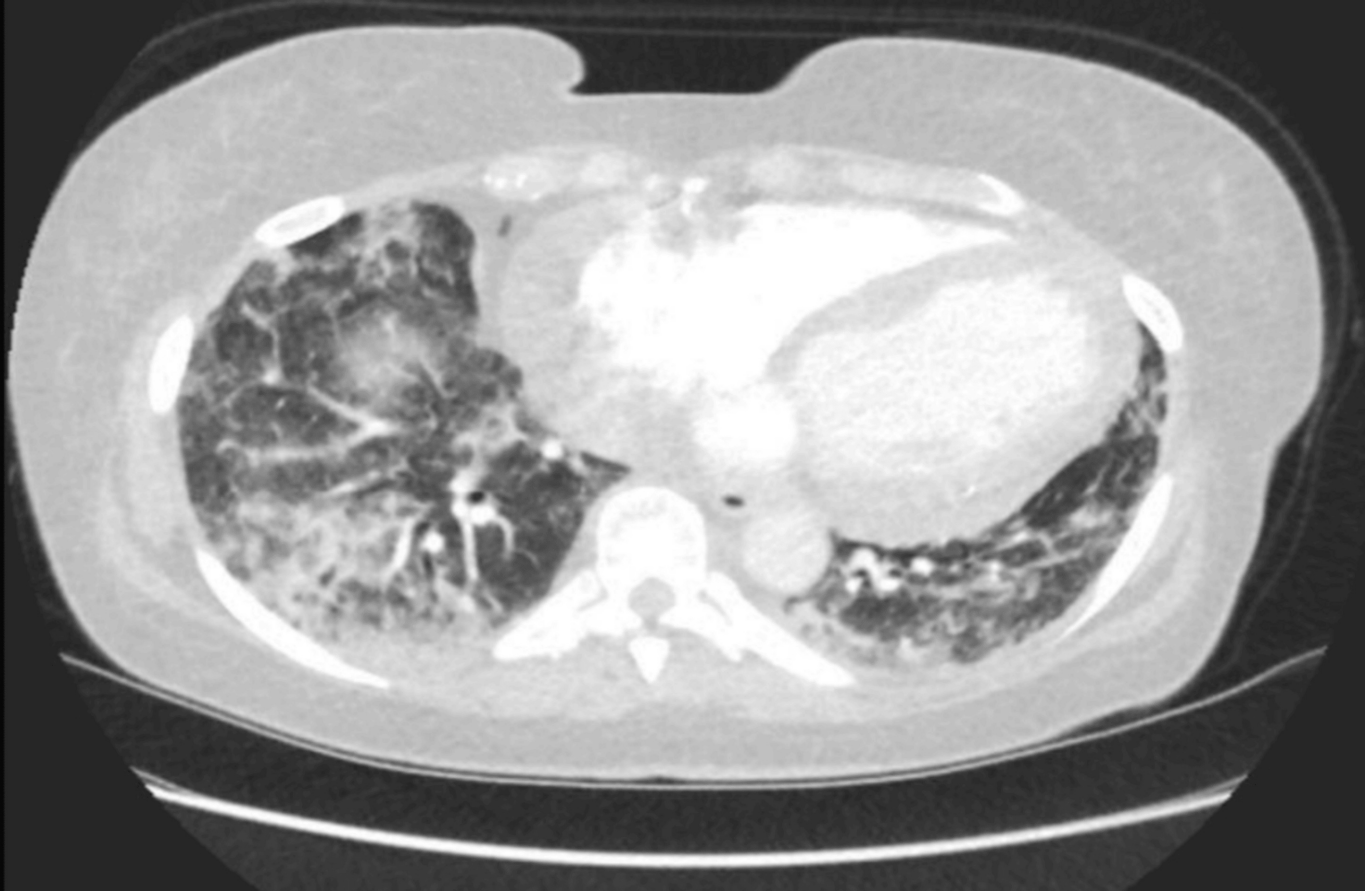
Basal lower lung zone view on CTPA showing a more severe form of patchy inflammatory changes.

A CT pulmonary angiography (CTPA) was done as well to rule out any possible embolism or infection as well and excluded pulmonary embolism and revealed patchy consolidation and ground-glass opacities with peribronchial sparing and subpleural bands, consistent with acute lupus pneumonitis (Figures 3-5).

Therapeutic intervention 

Then, following a rheumatology and respiratory MDT, she was commenced on treatment for lupus pneumonitis with high-dose intravenous methylprednisolone (250 mg stat, and then 1 g daily for the next three days), followed by oral prednisolone 40 mg od tapered with a consideration going on for commencing rituximab as well if symptoms did not stabilize. Empirical intravenous piperacillin-tazobactam was commenced as well due to concern for superimposed infection. She responded well to treatment, with her oxygen saturation improving to 96% and oxygen requirement reducing to 1 L/min. Her white cell count and C-reactive protein also improved, supporting both clinical and biochemical recovery.

**Figure 6:**
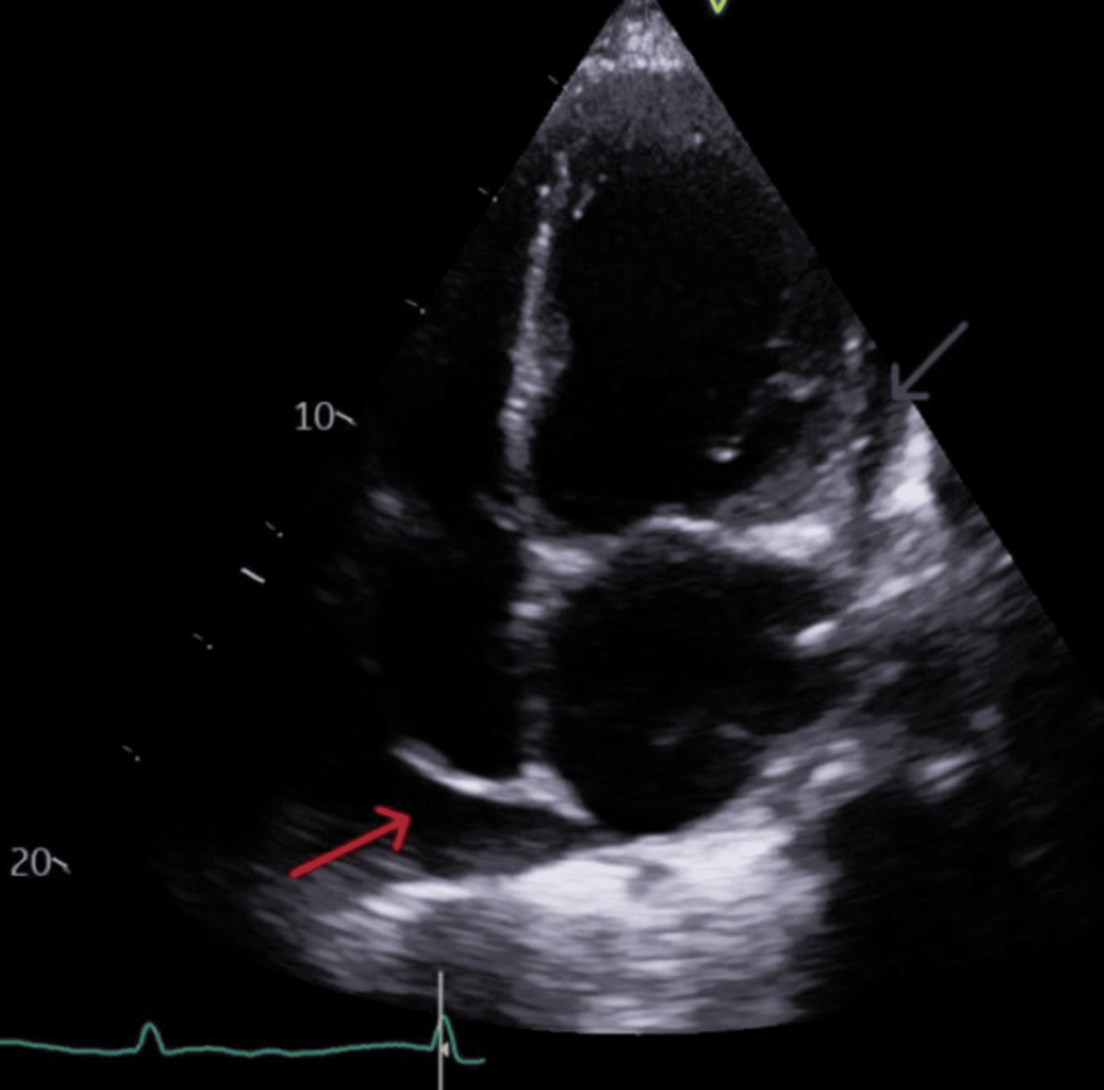
Echocardiogram: Modified apical four-chamber view. The red arrow depicts around 1.7 cm pericardial fluid collection around the right atrium (RA); the purple arrow shows smaller rim of pericardial collection around the left ventricle (LV). This all points towards evidence of polyserositis and reactive pericardial effusion.

A couple of days later, she developed a sudden increase in oxygen requirement to 4 L/min and became markedly breathless, complaining of mild chest pain. A transthoracic echocardiogram (Figure 6) was subsequently performed, which showed worsening heart failure with severe global systolic impairment (EF ~20%), impaired diastolic function with elevated filling pressures, moderate-to-severe low-flow aortic stenosis (AVA: 0.8 cm², DI: 0.32, mean gradient: 15 mmHg), mild-moderate aortic and mitral regurgitation, mild pulmonary regurgitation, and a small pericardial effusion (1.7 cm at RA) without tamponade.

**Figure 7:**
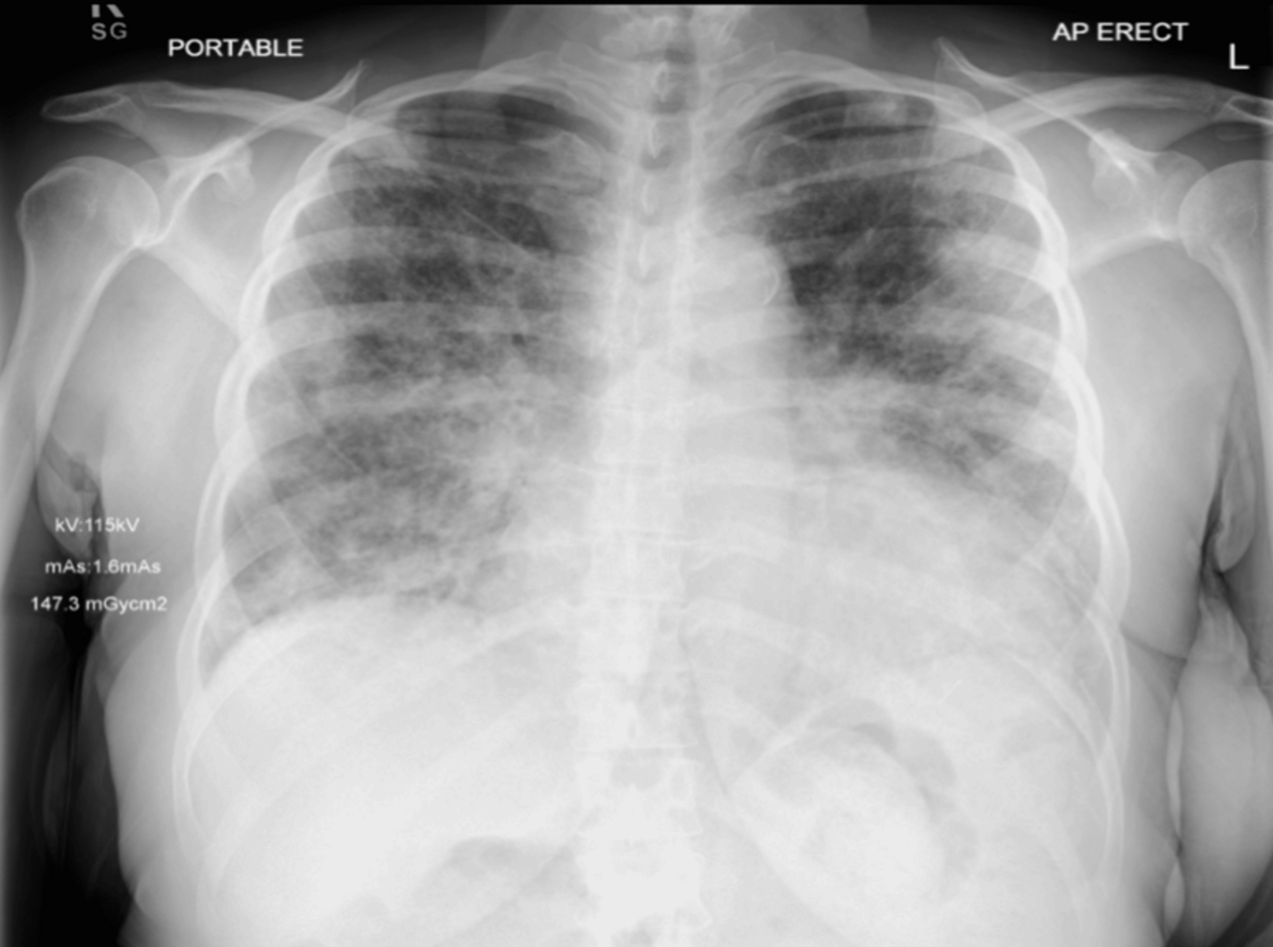
Chest X-ray demonstrating bilateral patchy and confluent opacities predominantly in the mid and lower lung zones, consistent with diffuse pulmonary edema—a radiographic feature of decompensated heart failure.

Rituximab was deferred due to the very low left ventricular EF (LVEF). During admission, whilst on oral steroids, she developed acute decompensated heart failure (Figure 7), requiring continuous positive airway pressure (CPAP) and intravenous furosemide infusion (160 mg/24 h, later reduced to 120 mg/24 h) with fluid restriction (1.5 L/day). This was complicated by stage 2 acute kidney injury, thought to be due to cardiorenal syndrome and diuretic therapy. Following renal optimization by discontinuation of nephrotoxic medications, and reduction of diuretic dose, both cardiac and renal functions improved. 

She subsequently stabilized, her oxygen requirement was weaned, and she was discharged home on a tapering oral steroid regimen and oral diuretics, with outpatient follow-up arranged under respiratory, rheumatology, and cardiology. This admission illustrates the multisystem complexity of systemic lupus erythematosus, with simultaneous pulmonary, cardiac, renal, and neurological involvement and highlights the critical importance of multidisciplinary management in patients with severe lupus flare.

## Discussion

Pulmonary involvement in SLE is well recognized, but ALP is a rare manifestation, affecting <5% of patients and associated with high mortality [4,5,7]. Typical presentation includes acute dyspnoea, cough, pleuritic pain, and hypoxia. Radiological findings often show bilateral ground-glass opacities and consolidation, overlapping with infection or heart failure, making diagnosis difficult [2,3].

ALP represents an immune-mediated inflammatory injury of the alveolar-capillary interface, driven by immune complex deposition and complement activation. Elevated levels of cytokines, such as IFN-γ, TNF-α, IL-6, IL-8, and IL-12, are frequently seen in SLE patients with pulmonary involvement [8,9,10]. Early inflammation results from alveolar and endothelial cell injury, releasing TNF-α, IL-1, and IL-8, which attract neutrophils that intensify tissue damage through NETosis [11]. Type I interferons further amplify this cycle by promoting autoantibody formation and sustaining inflammation, ultimately leading to fibrosis.

In our patient, the constellation of dyspnoea, pleuritic pain, hypoxia, and bilateral ground-glass opacities on CTPA, together with low procalcitonin (0.07 µg/L), normal complement levels (C3: 1.29 g/L, C4: 0.47 g/L), and mildly raised CRP (23 mg/L), favored ALP over infection [12-14]. These findings are consistent with prior studies showing that SLE patients with active disease may have normal or minimally elevated CRP and that raised procalcitonin (≥0.5 µg/L) strongly suggests bacterial infection rather than lupus activity [15].

Empirical broad-spectrum antibiotic therapy should be initiated immediately in suspected ALP to cover potential infectious triggers [16,10]. Aggressive immunotherapy should follow once infection is excluded-high-dose intravenous methylprednisolone, oral corticosteroids, intravenous cyclophosphamide, rituximab, plasma exchange, or IVIG can be considered depending on clinical response and comorbidities [10,17-19].

Our patient was appropriately treated with empirical intravenous piperacillin-tazobactam alongside pulse methylprednisolone (1 g × 3 days), followed by a tapering course of oral prednisolone. Rituximab was deferred due to severe left ventricular dysfunction, but her gradual improvement following corticosteroid therapy supports the diagnosis of ALP. Her management highlights the importance of early immunosuppression balanced against the risks of infection and cardiac decompensation, as well as the need for careful multidisciplinary coordination among rheumatology, respiratory, cardiology, and nephrology teams.

## Conclusions

This case illustrates the complexity of diagnosing and managing ALP in the context of multisystem lupus involvement. The overlapping clinical and radiological features with infection and cardiac dysfunction make early recognition particularly challenging. Our patient’s presentation emphasizes the value of integrating clinical judgment, targeted investigations, and multidisciplinary input in distinguishing ALP from other causes of respiratory failure. While immunosuppressive therapy was associated with clinical improvement in this instance, treatment outcomes cannot be generalized from a single case. Instead, this report reinforces the importance of vigilance for pulmonary manifestations in systemic lupus erythematosus and the need for individualized, evidence-informed management guided by careful exclusion of infectious and cardiac etiologies.
